# Rooting for nitrates: *ZmNLP3.2* positively regulates root biomass under low nitrogen conditions through *ZmAux/IAA14* inhibition

**DOI:** 10.1093/plcell/koae215

**Published:** 2024-07-24

**Authors:** Christian Damian Lorenzo

**Affiliations:** The Plant Cell, American Society of Plant Biologists; Center for Plant Systems Biology, VIB, Gent, B-9052, Belgium; Department of Plant Biotechnology and Bioinformatics, Ghent University, Gent, B-9052, Belgium

Plant roots are essential for resource uptake and anchorage to the soil. Root growth is dynamic and can be adjusted according to a plant's needs. For example, in the case of low nutrient availability, root growth can be promoted as a foraging response (Lynch 2019). Maize (*Zea mays*) possesses a primarily shoot-borne root system comprised of primary roots, seminal roots, crown roots, and lateral roots, which altogether contribute to successful plant growth (Hochholdinger 2009). Nitrogen (N) is an essential macronutrient for which sensing and uptake through the roots is partially regulated by NODULE INCEPTION-like proteins (NLPs). These NLPs bind nitrate and activate many downstream genes related to N signaling and assimilation (Cao et al. 2017). Despite their relevance, it remains unclear how NLPs contribute to root development mechanistically. Auxins are well-known hormones involved in almost all aspects of root growth and development (Roychoudhry and Kepinski 2022). However, no direct connection between NLPs and auxins in maize has been reported so far.

To unearth more about this intriguing process, recent work by Ruifeng **Wang and collaborators (Wang et al. 2024)** identified the NLP encoded by *ZmNLP3.2* and studied how it controls maize root growth. *ZmNLP3.2* was identified in a GWAS analysis comparing more than 300 inbred maize lines for root and shoot dry weight (RDW and SDW, respectively) under regular (Ctrl) or low N (LN) conditions. The group detected important SNPs that could be linked to a great percentage of the phenotypical variability among the lines closely flanking *ZmNLP3.2*. Haplotype analysis of the inbreds allowed to distinguish 2 specific groups: HAP1, which was associated with higher expression levels of *ZmNLP3.2* and presented higher RDW under LN; and HAP2, which showed the opposite pattern. Following this variation, the group developed both CRISPR-knockout (KO) and overexpressor (OE) lines and confirmed their previous observations, as *ZmNLP3.2* KOs displayed reduced RDW only under LN treatment while the OEs displayed increased RDW, SDW, and total dry weight, irrespective of the N condition. The *ZmNLP3.2* OE line also showed higher expression of N signaling genes and higher ammonium accumulation.

Digging deeper into how *ZmNLP3.2* promotes root growth under LN, the authors identified *ZmAux/IAA14* (known repressors of auxin signaling) as differentially expressed under different N conditions. They observed that *ZmAux/IAA14* expression decreased considerably upon LN in wild-type maize lines and increased or decreased significantly in *ZmNLP3.2* KO and OE lines, respectively. Moreover, *ZmAux/IAA14* KO and OE lines displayed opposite RDW and SDW phenotypes to those of *ZmNLp3.2* KOs and OEs. As Aux/IAA are generally regulated by *AUXIN RESPONSE FACTORS* (*ARFs*), the group performed yeast 1-hybrid assays and detected several maize ARFs that were able to bind to Aux/RE elements in the promoter of *ZmAux/IAA14.* Specifically*, ZmARF19* bound to and positively regulated *ZmAux/IAA14* expression, which the authors also confirmed by Chromatine inmuniprocipitation-qPCR (CHIP-qPCRs) and luciferase transactivation assays.

Finally, to understand how *ZmNLP3.2* affects *ZmARF19* action over *ZmAux/IAA14*, the group performed electrophoretic mobility shift assays and found that *ZmNLP3.2* weakened the binding of *ZmARF19* to regulatory sequences in the promoter of *ZmAux/IAA14.* Firefly/Renilla luciferase (LUC/REN) trans-activation assays using the *ZmAux/IAA14* promoter also displayed a 43% reduction in ZmARF19 binding to the promoter when *ZmNLP3.2* was coexpressed. In addition, virus-induced silencing of *ZmNLP3.2* in a *ZmAux/IAA14* OE background further decreased RDW values and led to even lower levels of ammonium accumulation under LN conditions than those of the OE lines alone. Considering these results, the authors put forth a possible model describing the mechanism behind the *ZmNLP3.2-ZmARF19-ZmAux/IAA14* regulation of root growth for the *ZmNLP3.2* HAP1 and HAP2 type inbreds (see Figure). In conclusion, this research sheds light on how NLPs regulate root growth through control of the auxin pathway to maximize nutrient uptake in limiting N conditions.

**Figure.**
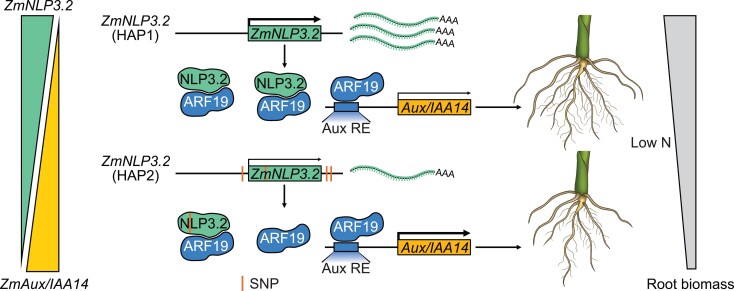
Action mechanism of *ZmNLP3.2* regulation over *ZmAux/IAA14* expression through *ZmARF19* interaction in HAP1 and HAP2 maize lines. Higher levels of *ZmNLP3.2* expression lead to reduced expression of *ZmAux/IAA14* and enhanced root growth. Reprinted from Wang et al. (2024), Figure 8.
